# Investigation of the Influence of Fiber Content, Processing Conditions and Surface Roughness on the Polymer Filling Behavior in Thermoset Injection Molding

**DOI:** 10.3390/polym15051244

**Published:** 2023-02-28

**Authors:** Ngoc Tu Tran, Andreas Seefried, Michael Gehde

**Affiliations:** 1 Department of Mechanical Engineering, Chemnitz University of Technology, 09126 Chemnitz, Germany; 2 Department of Mechanical Engineering, University of Transport and Communications (Trường Đại học Giao thông Vận tải), No.3 Cau Giay Street, Lang Thuong Ward, Dong Da District, Hanoi 100803, Vietnam

**Keywords:** thermoset molding compounds, injection molding, block flow, fountain flow, filler content, fiber orientation, processing conditions, surface roughness, wall slip

## Abstract

A completely opposite injection molding filling behavior of thermosets and thermoplastics by an effective and useful method developed by the authors was found. Specifically, for the thermoset injection molding, there is a strong slip between the thermoset melt and wall surface, which is not found for the injection molding of thermoplastic materials. In addition, the variables, such as the filler content, the mold temperature, the injection speed, and the surface roughness that could lead to or influence the slip phenomenon of thermoset injection molding compounds, were also investigated. Furthermore, microscopy was conducted to verify the correlation between the mold wall slip and fiber orientation. The results obtained in this paper open challenges in the field of the calculation, analysis, and simulation of mold filling behavior of highly glass fiber-reinforced thermoset resins in the injection molding process with consideration of wall slip boundary conditions.

## 1. Introduction

Materials composed of thermoset resins have special characteristics, such as high heat, chemical, and electric resistance. Moreover, glass fiber-reinforced thermoset injection molding compounds have a high strength and a low weight, making them an important substitute for thermoplastics and light metals [1]. Consequently, the demand for using thermoset resins is currently increasing. Thermoset polymer parts could be processed by various methods. One of them is the injection molding technology. Worldwide injection molding in the field of thermoplastics is an extremely common procedure. In contrast with thermoplastics, the method of thermoset injection molding has received much less attention, although it is used to produce excellent automotive products and electrical equipment, such as oil pumps, headlight boxes, and brake pistons [1].

The reasons for the thermoset’s low popularity could be found in the reactive nature of thermosets. In comparison to thermoplastics, for which viscosity only depends on temperature and shear rate, the viscosity of thermoset compounds also depends on the degree of cure [2]. In the processing of thermoset compounds, the viscosity curve results from two opposing effects. When the degree of cure is low, the viscosity of thermoset resins will decrease if temperature increases, which is the same for thermoplastic materials [3]. However, if the processing temperature reaches a certain level, the number of cross-linkings will increase rapidly. Hence, the degree of cure rises significantly, and it results in an increasing viscosity. Consequently, the viscosity curve of thermoset materials is characterized by a parabolic curve [4]. Additionally, the real curve progression depends on the degree of condensation and reactivity.

Although the injection molding “machine hardware” is nearly the same as for thermoplastics, the injection molding process of thermosets differs hugely from that of thermoplastics because of its complex viscosity behavior [5]. The widely explored and experienced area of thermoplastic injection molding uses temperatures for plasticizing from 150 °C to 300 °C. In contrast to these high temperatures, the mold requires cooling in a temperature range from 30 °C to 80 °C until the molding solidifies. Before being injected into the cavity, the thermoset injection molding compounds must be plasticized below cure temperature (maximum 100 °C) to reduce the risk of premature curing. After injection into the mold, thermoset materials require further heat to achieve final cure and solidification. Therefore, the thermoset injection mold reaches temperatures from circa 160 °C to 190 °C.

Due to the complex progression in the viscosity of thermoset polymer, some previous studies have been carried out regarding the injection machine, processing design, and processing characteristics of thermoset injection molding compounds. To avoid excess shear heating due to friction that leads to cure during the plasticizing zone, parameters, such as revolution speed, injection speed, as well as design elements, such as screw compressibility, screw point angle, or heating/cooling technology were studied [6,7,8,9,10]. As another aspect, the influence of the back flow of plasticized material was a wide exploratory topic. Other published investigations refer to the mold design and the product appearance by variation machine closing pressure, aeration in the mold, and draft of the ejectors [6,7,8,9,10]. However, these considerable investigations focused on the improvement of processing. The rheological mechanisms that form the basis of the flow behavior of thermoset injection molding compounds have not yet been inspected.

Some first experimental studies [11,12] about the mold filling behavior of thermoset injection molding compound were published. Through a glass plate that was inserted into the rectangular cavity, it was possible to observe the unsteady flow front of the thermoset polymer during the filling phase [12]. This observation represents progress in understanding the rheological characteristics of thermoset injection molding compounds during processing. However, an explanation was not found as to why the unsteady flow is visible. In addition, in order to protect the glass window, unrealistic low injection pressure parameters were used. Therefore, the experimental results have limited exploratory power for the flow behavior under high injection pressure conditions.

Regarding the mold filling behavior of thermoset phenolic resin under high pressure with various processing conditions, the gate-magnetization method developed by Yokoi and coworkers was applied to investigate the flow behavior of glass fiber (50%wt) reinforced phenolic resin compound inside a rectangular mold [13]. With the gate-magnetization method, magnetic particles are premixed into resin, and then the resin material is magnetized at the gate. A magnetic field is created at the gate of the mold, which magnetizes the magnetic particles. Magnetization timing is controlled by a computer. Three-dimensional flow patterns were analyzed by measuring the distribution of magnetized patterns at different sections of the molded part. The magnetized particles in the section were detected by using magneto detection liquid, which reacts to small amounts of magnetism. By observing the magnetized patterns it was found that a plug-like flow [11] builds up in the cavity in both the horizontal and vertical sections. A slip between the resin compound and the cavity wall occurred during the filling process. Especially in comparison to thermoplastic’s fountain flow behavior [14], the academic value of this new perception is huge. However, the magneto detection liquid can only react to small amounts of magnetism, which leads to difficulty in visual analysis of magnetized particles at different sections of a molded part. Furthermore, the explanation of the unsteady flow front was still unknown.

The mold filling behavior of highly fiber-reinforced phenol-formaldehyde resins was recently investigated under practical processing conditions, such as injection speeds and mold temperatures [15]. The experimental results show that there are two different areas on the surface of the incomplete part molded from 60% cavity volume. On the one hand, behind the melt front there is an area without the mold wall contact. The polymer melt in this area is less compacted, and the surface of the incomplete molded part is rough. On the other hand, near to the gate, there is an area with the mold wall contact. Consequently, the surface of the incomplete molded part in this area is smooth and the polymer melt is compacted. In addition, the length of the area with or without the mold wall contact is strongly dependent on the mold temperature and the injection speed. However, the underlying highly complex physical explanation of these appearances and the variations has not yet been interpreted. Furthermore, a technical method has not yet been developed to explain the unsteady flow front and the less compacted area.

Recently, further studies were performed by us to deal with the targeted investigation of the mold filling behavior of thermoset injection molding compounds, especially the slip phenomenon of the thermoset melt [16,17,18]. An effective and useful method, namely spotwise painting, was developed by the authors. This method was applied to analyze the slip phenomenon of the thermoset melt in the injection molding process. A series of incomplete molded parts were molded in the injection molding process. In the experiments, one high glass fiber-reinforced phenolic thermoset compound was studied with various mold temperatures and injection speeds.

For all investigated injection speeds and mold temperatures, the experimental results show that on the surface of the molded parts the white stripes appear clearly and that in the flow direction there is a movement of the white stripes. The slip phenomenon of phenolic polymer is believed to occur along the whole cavity because the phenolic polymer which is dyed white appeared even on areas which did not contact with the mold wall surface. In order to compare the movement of the phenolic melt and thermoplastics melt on the cavity surface, the spotwise painting was also applied to thermoplastic injection molding (PA6 with 30% of glass fiber of BASF Ultramid^®^). The experimental results show an opposition in the mold filling behavior of thermosets and thermoplastics polymer on the cavity surface in the process of filling a mold. For phenolic thermoset injection molding, there is a strong slip between the phenolic melt and wall surface, which is not found for the injection molding of the PA6 with 30% glass fiber [16,17,18]. The factors which could lead to these differences are not only the influence of resin or high mold temperature, but also the amount of fiber and filler. These opposite results help us to conclude that it is impossible to apply the thermoplastics injection molding concepts and rheological theory or simulation packages, respectively, for thermosets.

The mechanism of a strong slip on the interface between the phenolic melt and the mold wall surface was observed. At the beginning, the compound does not have contact to the wall surface, so there is no slip between melt and wall. If contact occurs, the slip phenomenon starts. The visible movement of the painting shows that the velocity of the phenolic melt near to the wall surface is above zero. As such, the mold filling behavior as a plug shear flow is established. However, other variables, such as the amount of filler, the mold surface roughness, the mold temperature, and the injection speed, which can lead to or influence the slip phenomenon of phenolic thermoset injection molding compounds in the filling phase of the injection molding process, have not yet been investigated. In addition, the influence of wall slip on the fiber orientation in the filling phase of the injection molding process has also not yet been analyzed.

In the present article, the developed effective and useful method, namely, the spotwise painting of the mold wall surface, was continually applied to study the effect of filler content, the processing condition, such as the mold temperature and the injection speed, and the surface roughness on the polymer filling behavior in the thermoset injection molding process. Microscopy was conducted to verify the correlation between slip and fiber orientation. The received results of this research will open new study ideas in the field of thermoset injection molding, both experimentally and in simulations.

## 2. Materials and Methods

### 2.1. Material and Equipment

Three commercial thermoset phenolic injection molding compounds with different filler content for the injection molding process were selected and ordered from a material supplier. The filler content is from 55% to 80%, as shown in Table 1.

**Figure 1:**
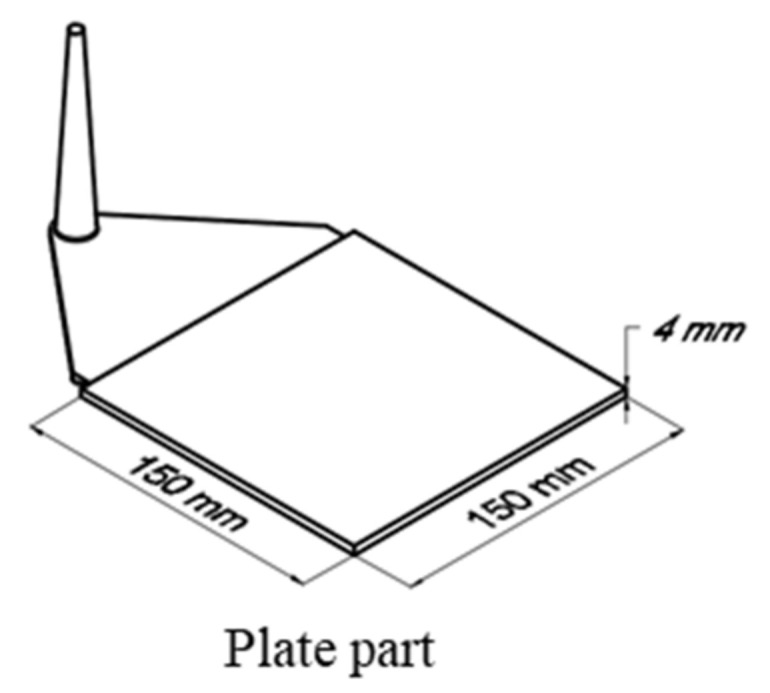
Studied object.

**Figure 2:**
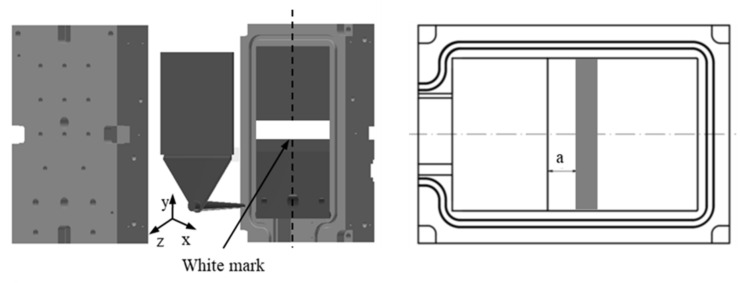
A simple standard two-plate mold and schematic location of white marks on the mold wall surface.

**Figure 3:**
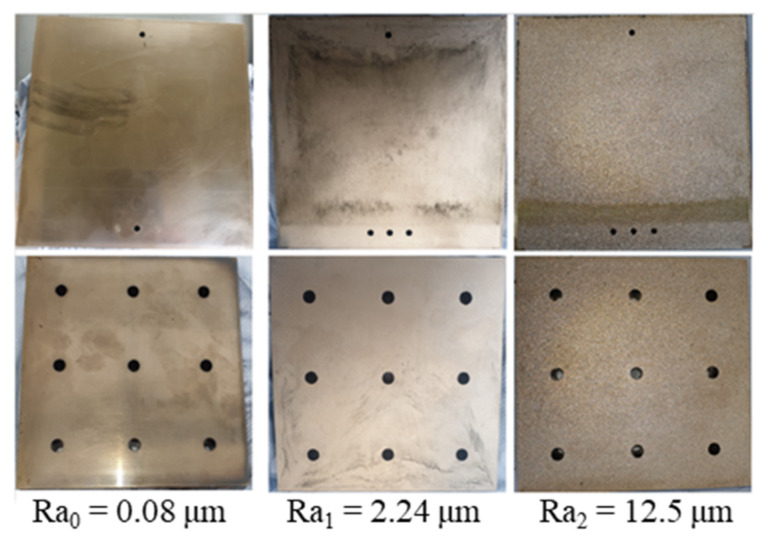
The cavity inserts with different surface roughness.

A hydraulic Krauss Maffei injection molding machine KM 150-460B, with a screw diameter of 45 mm and a three-zone plasticizing cylinder, was employed. The study object (Figure 1) was a plate with the dimensions of 150 mm × 150 mm × 4 mm. A simple standard two-plate mold (Figure 2) with three different values of cavity surface roughness was used. The values of surface roughness (Figure 3) are Ra_0_ = 0.08 μm, Ra_1_ = 2.24 μm, and Ra_2_ = 12.5 μm, respectively.

The effective and useful method, namely the spotwise painting of the mold wall surface developed by authors, was continually applied to study the mold filling behavior of thermoset injection molding compounds. To ensure the homogenous and high qualitative application of the color mark, a white printing-ink from Plastikote was selected. In addition, the rectangular stamp equipment for the constant color application was used.

### 2.2. Experimental Procedure

In order to investigate the variables, such as the amount of filler, the mold surface roughness, the mold temperature, and the injection speed that can lead to or influence the slip phenomenon of thermoset injection molding compounds in the filling phase, in each injection molding process of the following procedures, the white mark was painted on the constant position of the mold wall surface. The schematic position of the constant rectangular white mark which was painted on the wall surface of the mold is shown in Figure 2. The distance a of 20 mm between the location of the white mark and the boundary line between the cavity and the film gate is constantly kept in all experimental processes.

#### 2.2.1. Investigation of the Influence of Filler Content on Slip Phenomenon

To analyze the influence of different fiber content on the slip phenomenon between thermoset melt and the wall surface, injection molding experiments of three chosen phenolic injection molding compounds were conducted under constant processing conditions. The temperature profile in the injection chamber (cylinder temperature) is 100 °C-80 °C-60 °C that is constantly kept for each injection molding process of all of the following procedures. The injection speed is 16 cm³/s, and the mold temperature is 175 °C. In this step, the simple standard two-plate mold with surface roughness Ra_0_ was used. The effect of filler content on the slip phenomenon was explained via analysis of the movement of the white marks on the surface of molded parts. 

#### 2.2.2. Investigation of the Influence of Injection Speed and Mold Temperature on Slip Phenomenon

Based on the analysis of the movement of the white marks on the surface of the molded parts in Section 2.2.1, the thermoset injection molding compound with the weakest or the strongest slip phenomenon was found. The thermoset injection molding compound with the strongest slip phenomenon was selected to perform further injection molding experiments. To investigate the influence of injection speed on the slip phenomenon, the experimental injection molding processes were firstly started with a constant mold temperature of 175 °C and different injection speeds of 8 cm³/s, 16 cm³/s, and 32 cm³/s, respectively. After that, to analyze the influence of mold temperature on the slip phenomenon, the other experimental injection molding processes were performed with a constant injection speed of 16 cm³/s and different mold temperatures of 160 °C, 175 °C, and 190 °C, respectively. The simple standard two-plate mold with surface roughness Ra_0_ was used in all processing conditions.

The effect of injection speeds and mold temperatures on the slip phenomenon was also explained via analysis of the movement of the white marks on the surface of molded parts. Furthermore, in order to have more information for analyzing the mold filling characteristics of the chosen thermoset injection molding compounds, a series of incomplete molded parts with different percentages of cavity volume were molded in the experimental injection molding process.

#### 2.2.3. Investigation of the Influence of Surface Roughness on Slip Phenomenon

The phenolic injection molding compounds with the weakest and strongest slip phenomenon, as shown in Section 2.2.1, were selected to conduct injection molding experiments. The simple standard two-plate mold with three surface roughness, namely Ra_0_, Ra_1_, and Ra_2_, were employed. The injection speed was 16 cm³/s, and the mold temperature was 175 °C.

#### 2.2.4. Investigation of Slip Phenomenon on the Fiber Orientation

**Figure 4:**
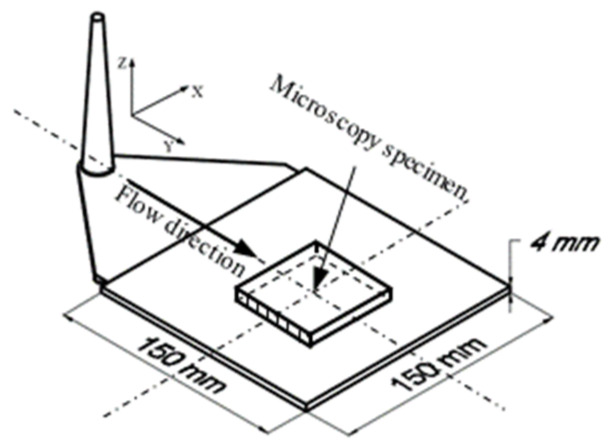
Position of specimens for analyzing fiber orientation.

Microscopy was conducted to verify the correlation between slip and fiber orientation. The microscopy specimens with dimensions of 20 mm × 20 mm × 4 mm (Figure 4) were prepared from the injection molded parts to detect the relationships between the layer thickness of equally oriented fibers and the intensity of the slip phenomenon.

## 3. Results and Discussion

### 3.1. Influence of Filler Content on Slip Phenomenon of Highly Filled Thermoset Molding Compounds

**Figure 5:**
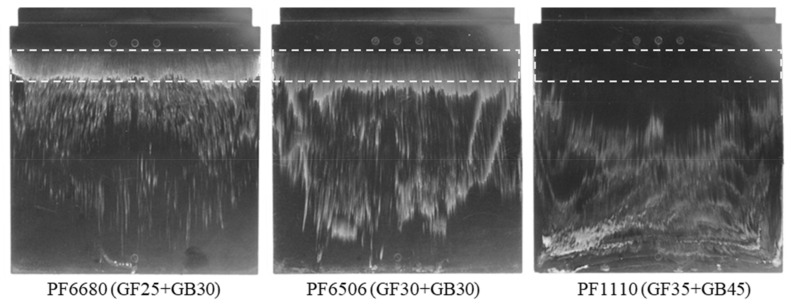
Influence of filler content on the slip phenomenon.

Figure 5 shows that there is movement of the polymer dyed white color on the surface of the molded parts from the original painted position (rectangular shape) to the end of the molded part. The cause which leads to the movement of the white color is the slip phenomenon on the interface between the phenolic polymer and the mold wall surface. However, the intensity of the white stripes on the surface of the molded parts is not the same. It is dependent on the amount of filler. Specifically, with PF6680 (25% of short glass fiber and 30% of glass ball), there is still the white color at the original painted position. However, with PF6506 (30% of short glass fiber and 30% of glass ball), the white color density at the original painted position is not clear as with PF6680. In contrast to PF6680 and PF6506, with PF1110 (35% of short glass fiber and 45% of glass ball), the white color does not appear at the original painted position. This means PF1110 (GF35+GB45) has the strongest slip phenomenon, followed by PF6506 (GF30+GB30) and PF6680 (GF25+GB30). Therefore, it could be concluded that the filler content is the main reason for the wall slip phenomenon on the interface between the phenolic polymer and the mold wall surface. As the amount of filler increases, the wall slip phenomenon is stronger.

### 3.2. Influence of Injection Speed and Mold Temperature on Slip Phenomenon

**Figure 6:**
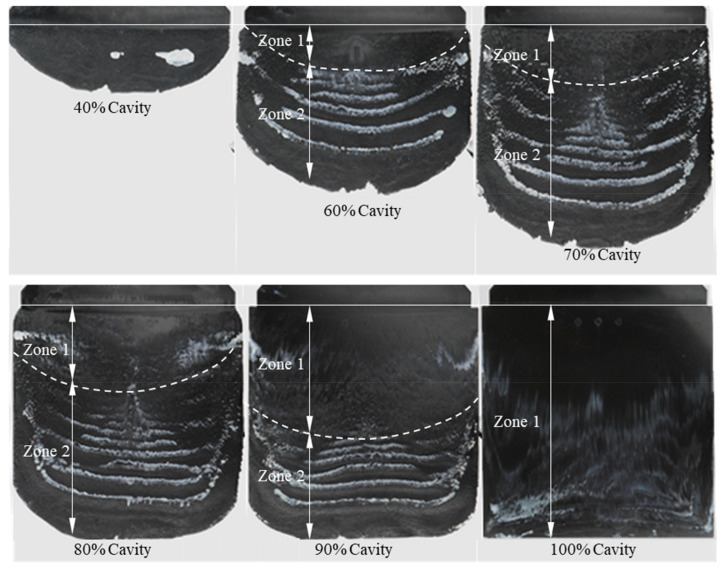
Mechanism of slip phenomenon. Material is PF1110 (GF35+GB45). Mold temperature is 175 °C, and injection speed is 16 cm³/s.

The thermoset injection molding compound PF1110 (35% of short glass fiber and 45% of glass ball) with the strongest slip phenomenon was selected to carry out further injection molding experiments under various injection speeds and molding temperatures. The mechanism of slip phenomenon is shown in Figure 6. Starting with the incomplete part that was molded from only 40% cavity volume, the surface of this molded part is rough because the phenolic melt has interrupted contact with the mold wall surface. The white color starts appearing on the surface of this molded part. However, the phenolic polymer region dyed white looks like small dots instead of the original rectangular white mark which has already been painted on the wall surface of the mold. Furthermore, the location of white dots on the surface of this molded part is exactly in the area of the original rectangular white mark painted on the mold wall surface. Therefore, there is not yet any movement of the white stripe on the surface of this incomplete molded part, which means that the phenolic melt had no contact with the wall surface at the beginning of flow. The white stripes appear clearly on the surface of the incomplete parts molded from cavity volumes of 60%, 70%, 80%, and 90%, respectively. Furthermore, it could be seen in this molded parts that there is strong movement of white stripes in the flow direction. The length of the phenolic polymer region which was dyed white does not reach the melt front. The intensity of the white stripes decreases from the location of the original white mark to the melt front. Specifically, the white color seems to disappear on the area that has contact with the mold wall surface where the original rectangular white mark was applied. Based on the surface roughness of the incomplete molded parts and the distribution of the white stripes, the incomplete molded parts are classified into two different zones. Zone 1 is located next to the gate. Zone 2 is located next to zone 1 and behind the melt front. Beginning with zone 1, the thickness of the molded parts is 4 mm. The polymer is properly compacted and has complete contact with the wall surface so that the surface of the molded parts in this zone looks very smooth. The intensity of the white stripes in this zone is approximately the same. In contrast, with zone 2, the polymer is less compacted than in zone 1, especially near to the melt front. The thickness of the incomplete molded parts in zone 2 is approximately from 3.5 mm to 4 mm, so that there is interrupted contact between the polymer and the mold wall surface. Consequently, the surface of the molded parts in zone 2 looks very rough. Next to the melt front, the polymer without the white stripes is found, which seems to show that this polymer region originated from the initial polymer portion which flew into the cavity before reaching the white mark. The length of zone 1 increases when increasing the injection cavity volume. As the cavity volume is filled with 100% of the phenolic polymer, zone 2 disappears and the white color density near to the end of complete molded part is higher than the density of the white color near to the gate.

**Figure 7:**
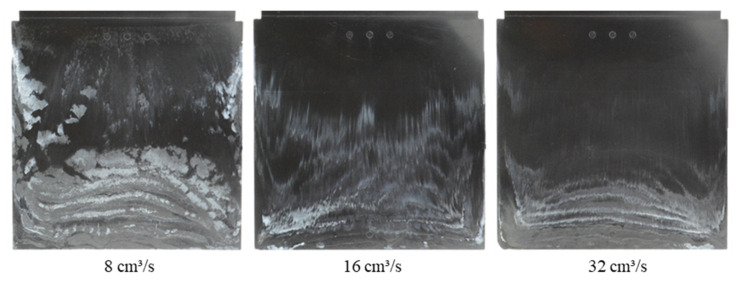
PF1110 (GF35+GB45); influence of injection speeds on the slip phenomenon.

**Figure 8:**
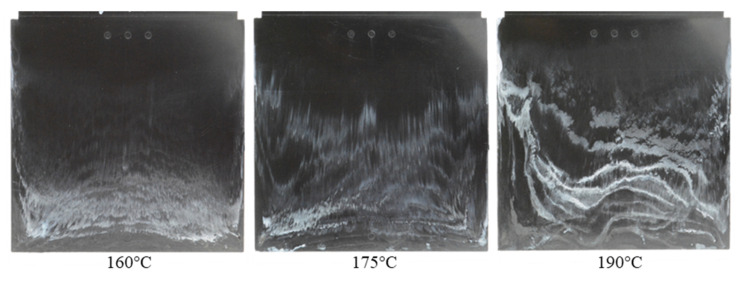
PF1110 (GF35+GB45); influence of mold temperatures on the slip phenomenon.

With different injection speeds and mold temperatures, it was found that the white stripes appear clearly on the surface of the molded parts and there is a slip of the white stripes in the flow direction (Figure 7 and Figure 8). However, the white color density on the surface of molded parts depends on the injection speed and the mold temperature. With the constant mold temperature of 175 °C and the different injection rates of 8 cm³/s, 16 cm³/s and 32 cm³/s (Figure 7), it could be seen that when the injection speed increases, the wall slip phenomenon on the interface between the phenolic polymer and the mold wall surface is stronger. With the constant injection speed of 16 cm³/s, the degree of slip phenomenon between the thermoset melt and the wall surface decreases as the mold temperature increases, as shown in Figure 8.

**Figure 9:**
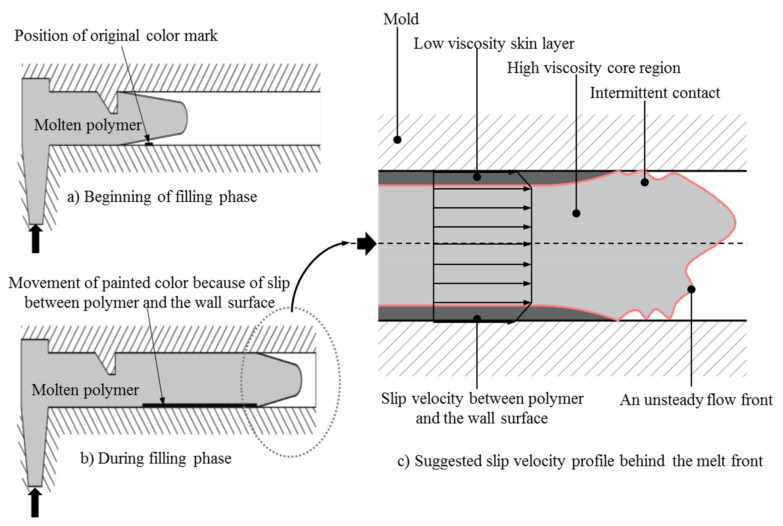
The theoretical foundation of slip phenomenon between the thermoset melt and the mold wall surface.

Based on the experimental results, the theoretical foundation of the slip phenomenon of thermoset injection molding compounds in the filling phase of the injection molding process is supposed, as shown in Figure 9. At the beginning of the filling phase, the thermoset injection molding compounds does not have contact with the wall surface, so there is no slip between thermoset melt and wall surface. The slip phenomenon starts immediately when the thermoset melt has first contact with the mold wall surface contact. The degree of slip velocity between the thermoset melt and the mold wall surface is strongly dependent on the injection speeds and the mold temperatures. Specifically, before being injected into the cavity, the thermoset injection molding compounds are plasticized in the screw chamber with the cylinder temperature profile of 100 °C-80 °C-60 °C. Therefore, the thermoset melt temperature is about 100 °C at the beginning of the filling process. During the filling of the cavity, the temperature of the thermoset melt increases because of heat transfer from the hot mold to the thermoset melt. Consequently, the thermoset melt temperature near to the mold wall surface is higher than in the core region. The temperature of thermoset melt located near to the mold wall surface could reach the mold temperature in the filling phase. However, the temperature of the thermoset melt in the core area is much lower than the mold temperature. Furthermore, the time for filling the cavity completely is very short. For example, when the injection speed is 16 cm³/s and the mold temperature is 175 °C, the filling time is only 8 s. As result, the degree of cure in the filling phase is very low. Therefore, the viscosity of the thermoset melt in the filling phase depends strongly on the shear rate and temperature. The influence of the degree of cure on the viscosity behavior of thermoset melt could be neglected. There are two different viscosity layers over the cross section of the cavity, as shown in Figure 9. Near to the mold wall surface, the viscosity of the thermoset melt is low because of the high temperature. In contrast, in the core region, the temperature of the thermoset melt is low, which decreases the viscosity of the thermoset melt. When the mold temperature is kept constant and the cavity of the mold is filled with the higher injection speed, the shear rate of the thermoset melt in the filling phase is higher, leading to a significant decrease in the viscosity. As a result, the degree of slip velocity between the thermoset melts and wall surface is stronger. In the case of decreasing the mold temperature while the injection speed is kept constant, the viscosity increases significantly. Consequently, the degree of slip velocity between the thermoset melt and the wall surface is weaker.

### 3.3. Influence of Surface Roughness on Slip Phenomenon

**Figure 10:**
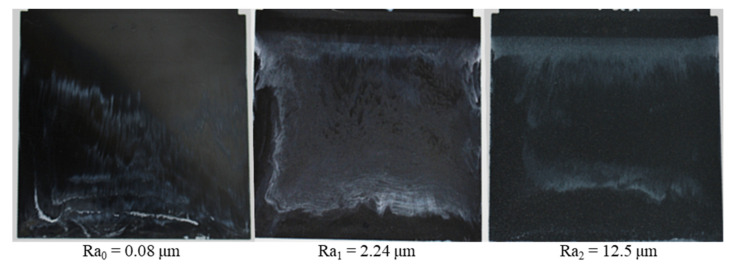
PF1110 (GF35+GB45); influence of surface roughness on the slip phenomenon.

**Figure 11:**
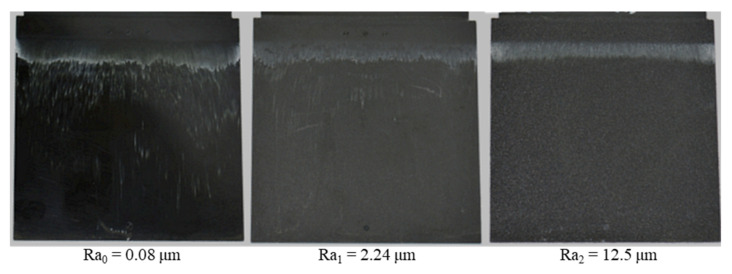
PF6680 (GF25+GB30); influence of surface roughness on the slip phenomenon.

As shown in Section 3.1 and in Figure 5, PF6680 (GF25%+GB30%) has the weakest slip phenomenon and PF1110 (GF35+GB45) has the strongest slip phenomenon. These materials were selected to conduct injection molding experiments with three surface roughness values, namely Ra_0,_ Ra_1_, and Ra_2_. The injection speed was 16 cm³/s and the mold temperature was 175 °C, which was also used to investigate the influence of filler content on the mold filling behavior of thermoset injection molding compounds. The movement of polymer which was dyed white color on the surface of molded parts from the original painted position to the end of the molded part is strongly dependent on the material and the surface roughness, as shown in Figure 10 and Figure 11. For both materials, the degree of slip phenomenon decreases when the surface roughness increases. The experimental results of PF6680 show this with the surface roughness of Ra_1_ and Ra_2_, as the location of the white stripes is exactly the same before and after injection molding. This means there is no slip of the white stripes.

### 3.4. Influence of Slip Phenomenon on the Fiber Orientation

**Figure 12:**
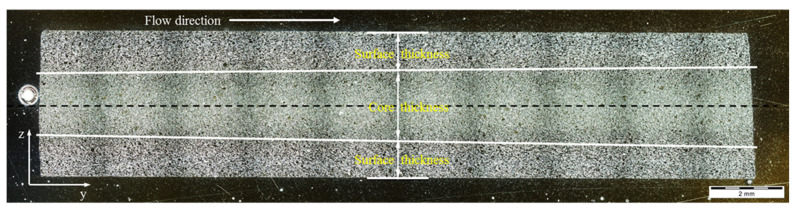
Fiber oriented under the surface of the molded part.

**Figure 13:**
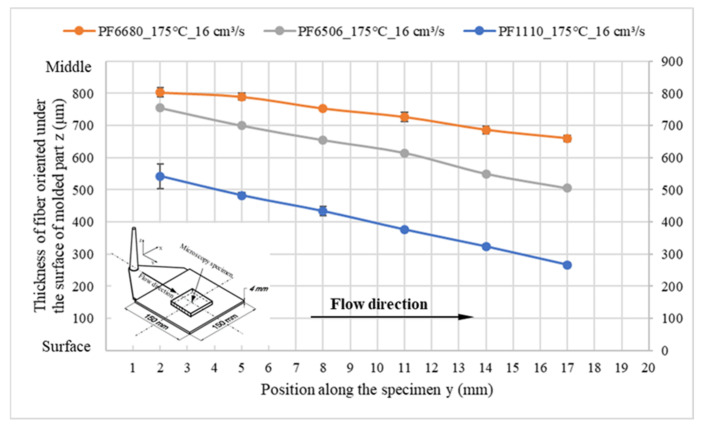
Influence of wall slip on the thickness of fiber oriented under the surface of the molded part.

In spite of having a strong slip between the thermoset melt and wall surface, fiber in the flow direction is still oriented under the surface of the molded parts. With all the investigated processing conditions and surface roughness, it was found that the flow direction of the oriented fiber under the surface of the molded part decreases, as shown in Figure 12. However, the thickness of the oriented fiber in the flow direction is strongly dependent on the degree of slip phenomenon. Despite the fiber amount of PF6680 being only 25%, which is the lowest compared with PF6506 (GF30%) and PF1110 (GF35%), the thickness of the oriented fiber under the surface of the molded part from PF6680 is two times higher than PF1110, as shown in Figure 13. The main reason for this difference is the degree of wall slip phenomenon that is shown in Figure 5. In the filling phase, PF6680 has a weaker slip phenomenon than PF1110.

**Figure 14:**
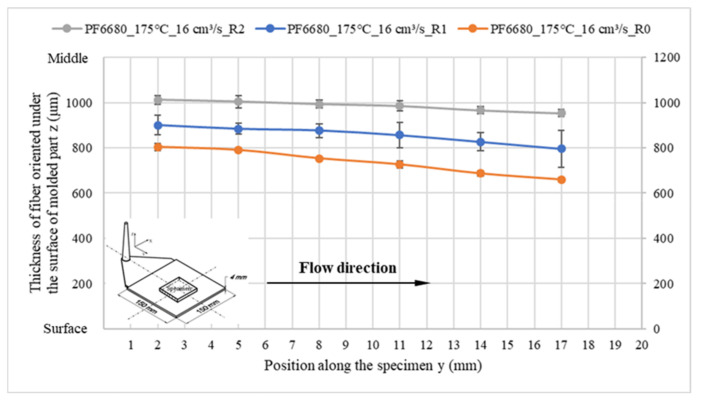
PF6680 (GF25+GB30); the influence of surface roughness on the thickness of the fiber orientation under the surface of molded part.

**Figure 15:**
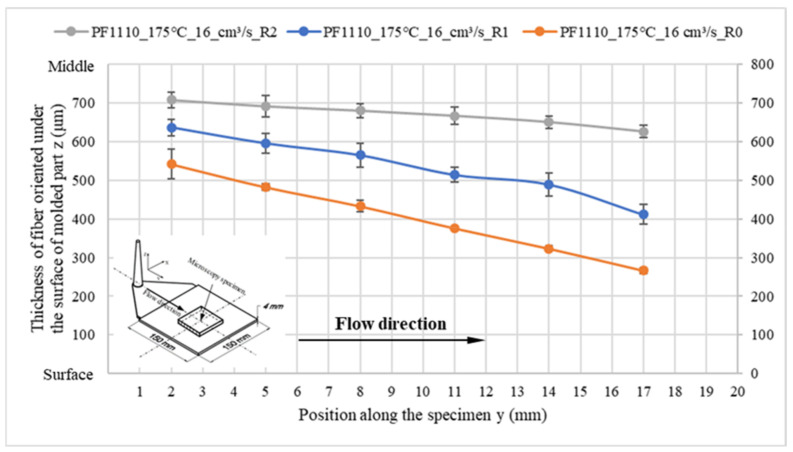
PF1110 (GF35+GB45); the influence of surface roughness on the thickness of fiber orientation under the surface of molded part.

**Figure 16:**
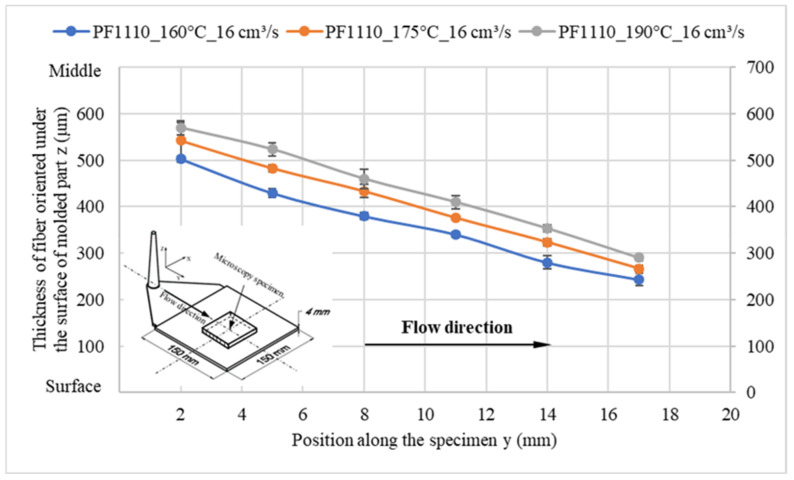
PF1110 (GF35+GB45); the influence of mold temperature on the fiber orientation under the surface of molded part.

**Figure 17:**
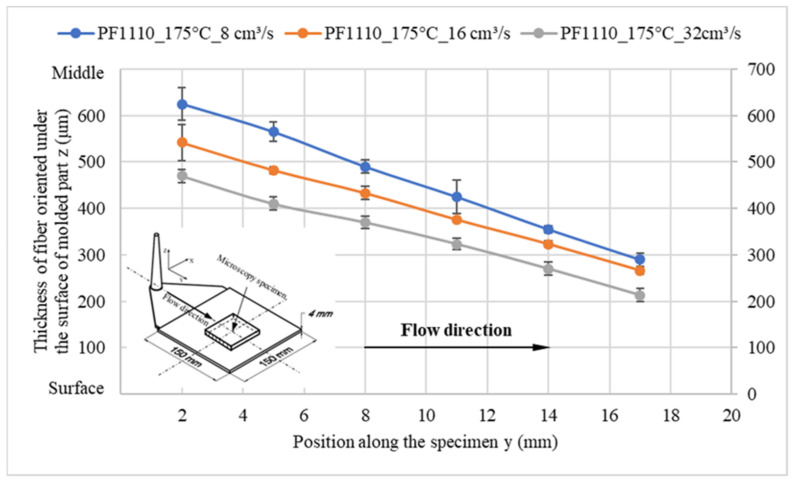
PF1110 (GF35+GB45); the influence of injection speeds on the fiber orientation under the surface of molded part.

When the surface roughness of the cavity increases, it decreases the degree of wall slip phenomenon, and the thickness of the oriented fiber under the surface of these molded parts significantly increases, as shown in Figure 14 and Figure 15. Finally, the microcopy specimens of parts molded from material PF1110 under different injection speeds and mold temperatures were used to analyze the fiber orientation. All results (Figure 16 and Figure 17) show that the higher injection speed and lower temperature increase the wall slip phenomenon, which leads to a decrease in the thickness of fiber orientation under the surface of the molded parts.

## 4. Conclusions

The mold filling behavior of the phenolic polymer on the cavity surface during the process of filling a rectangular cavity was successfully investigated by painting the mold wall surface with the rectangular white mark. For the whole investigation, it was found that the slip phenomenon of phenolic thermoset injection molding compounds is strongly dependent on the filler amount, the injection speed, the mold temperature, and the surface roughness. A lower filler amount and injection speed and a higher mold temperature and surface roughness decrease the wall slip phenomenon of the thermoset melts. Consequently, the thicknesses of the oriented fiber under the surface of molded parts increase significantly. The received results are background knowledge for subsequent studies in the field of thermoset injection molding, such as the theoretical foundations of the calculation, analysis, and simulation fiber orientation in the filling phase of the injection molding process with consideration of the wall slip phenomenon.

## Figures and Tables

**Table 1:** Experimental materials.

Abbreviation	Commercial Name	Manufacturer
PF-GF25+GB30	Bakelite PF6680	Bakelite
PF-GF30+GB30	Bakelite PF6506	Bakelite
PF-GF35+GB45	Bakelite PF1110	Bakelite

## Data Availability

The data presented in this study are available on request from the corresponding author.
